# Supplementary material for: Vocalization with semi-occluded airways is favorable for optimizing sound production
Source: PLoS Comput Biol. 2021 Mar 29;17(3):e1008744. doi: 10.1371/journal.pcbi.1008744 (PMC8031921; doi:10.1371/journal.pcbi.1008744)
Supplement: Attachment — Submitted filename: Reply to Reviewers_PLOS comp biology.docx [file pcbi.1008744.s001.docx]

Alison Marsden, Associate Editor

Natalia Komarova, Deputy Editor

PLOS Computational Biology

Dear Editors

Thank you for the reviews of the manuscript "VOCALIZATION WITH SEMI-OCCLUDED AIRWAYS IS FAVORABLE FOR OPTIMIZING SOUND PRODUCTION". They are highly appreciated. Below are our responses to all comments and suggestions made by the three reviewers.

Note 1: Reference to line numbers apply to the market-up revised copy, not the clean copy.

Note 2: The author Kevin Cox asked to be removed because the data from his dental lab were not used in the second submission of the paper.

Reviewer #1:

Abstract: This sentence is not clear: “It is hypothesized that vocalization with a semi-occluded airway has an overall widening effect on the airway” and suggests that the act of semi-occluding is to widen the airway (I am assuming that is not what is meant)

Response: Sentence revised to include “pressure” as the widening effect, lines 46-47

L. 58: Plural needed – ‘received by listeners’ (unless there is a reason that I have not spotted to be singular?

Response: Corrected, lines 58-59

L. 61: The comparison with sounds radiated from the skin seems out of place here – or I am missing the point?

Response: Sentence revised to clarity radiation from mouth openings versus skin surfaces, lines 62-64

L62: This sentence is also a bit of a non-sequitur: narrow the tract, sound is more efficient (OK) then you can open the mouth (after practice). This implies (to me at least) that practice can obviate any advantage of a narrowed tract.
Response: Sentences revised to clarify “before” and “after” effects, lines 66-69

L. 64: Here there is a jump from the ‘narrow tube at the mouth’ to the ‘airspace directly above the vocal folds’ – another non-sequitur to me. How far up the tract is ‘directly above’ the vocal folds – the last sentence indicates that this allows a large variety of sounds; I assume not all the vowels etc.?
Response: Directly above means the epilaryngeal airway. There should be no limitation on vowel production, lines 67-69

L. 88: Is there evidence for ‘optimally’ to support the statement after ‘presumably’? I do not see that the semi-occluded tract exercises proves that this statement is supported: ‘optimal airway configuration and optimal posturing and shaping of the vocal folds for vibration’.
Response: Less effortful and more efficient has been used to replace optimal, line 94

L. 92: For no brass players a reference for ‘the variable interaction experienced by brass instrument players who mute the instrument at the bell’.
Response: A reference to Baines (1992) has been added, line 100.

L. 103: The word ‘sharp’ here is undefined and could be a hostage to fortune – isn’t ‘square or saw-tooth-like’ sufficient; indeed, might ‘pulse’ be more appropriate than ‘square’?
Response: Sharp has been deleted and replaced with square, pulse, and sawtooth-like, lines 111-112

L. 106: I think something should be added to exemplify what the ‘acoustic interaction between ..source increases’ means in practice – this paper will be of great interest to singers.
Response: The beginning paragraph on interaction has been revised, lines 115-121

L. 110: What is the relationship between impedance and source vibration frequency and how might it affect the system?
Response: The relation between *f*_o_ and the airway impedance has been clarified, lines 121-124

L. 109: Is there any condition on which ‘portion’ of the airway might be narrowed in terms of the change in impedance?
Response: The condition ”below the first resonance” has been added, line121

L. 119: Just to be clear, state what sound was phonated into a drinking straw in Laukkanen’s experiment.
Response: No vowel or tongue intentionally change when phonating into tube, clarified on lines 132-134

L. 133: A bit of a non-sequitur here – on the one hand humans do not use widening much but when we need to we can widen the tract (deep breathing is an interesting case given the breath control care that singers employ so I wonder if this leads to more tract widening strategies arising from more conscious breath control?).
Response: The deep breath in singing has been added to the argument, lines 147-148

L. 169: Justify the decision to use 107 short circular sections and why an 8 cm tube at the lips.
Response: More detail has been given for section lengths and number of sections, lines 184-192

Figure 3: It would be useful to have a few landmarks indicated on the diagram to aid readers’ geography such as the trachea, glottis, velum, lips, nasal tract.
Response: The figure was updated with labels.

L. 169 and L. 179 differ in the length quoted for the flow resistant tube (8 cm and 10 cm respectively) – cannot both be right!
Response: 10 cm is the correct length, as indicated in the Fig. 3 caption

L182. I would say that 300 Hz is in the middle of a typical singing range - should be made clear it is not for speech range.
Response: Sentence revised to include the middle of the singing range, line 204 - 206

L182. Brackets do not close.
Response: Done

L183. Reference or show the obtaining of this frequency.
Response: The frequency calculation is described in the Methods section, which is clarified on lines 207-210

L.185. Define clearly the meaning of ‘post-pressure’.
Response: “Pre: and “post” pressure have been defined more clearly on lines 211-213

P.190. Make clear exactly where the nearly doubling is located – it is not clear to me by eye on the figure. Is it the fact that it is near doubling that is crucial or just the fact that there is a significant expansion?
Response: The location along the airway is specified at 18.4 cm, lines 218-219. The doubling is not crucial and will vary with lung pressure and oral tube resistance.

Figure 5: I note that these effects are plotted for 2.5 kPa lung pressure – how do they vary across typical lung pressure ranges (if that can be easily added) – this would place this plot in a useful place for practitioners and at the very least would benefit from a comment I think.
Response: The expansions vary linearly with pressure in the model, but would eventually saturate due to nonlinear wall stiffness, which has not been quantified. An entire paragraph has been added to explain this, lines 242-246

L. 233: Remove ‘that’.
Response: Done

Table 1: What are the error margins in these measurements? Line 238 indicate that the cross-sectional area measurements were “very difficult to obtain accurately” without quantifying the difficulty.
Response: The measurements were order-of-magnitude because they were obtained from 2D images whose planes generally did not line up with the plane normal to the airway axis, lines 287 - 298. It is not possible to quantify the difficulty solely from the images.

Figure 6: I find the plotted points confusing – there are 6 plotted points for each of three phonation types that are measured from 8 subjects (males and females – 4 each I assume) from whom the data are averaged phonating at three loudness conditions. So are the 6 points 3 loudness levels from males and females – I think reading loudness from the plot should be very clear in the figure caption so the points can be unambiguously identified (unless I have not understood it).
Response: The plot has been improved to include a color scheme for loud, medium, and soft, which is also described in the figure caption.

L. 292: Noted that the sub glottal measurements were taken from Story et al, but it is not clear that the modelled measurements were appropriate tract dimensional matches (which I assume they were).
Response: More clarity is provided on where subglottal and supraglottal shapes were obtained, lines 335-336.

Fig. 7 – a lovely result.
Response: Thank you

Fig. 8: Make it clear where the 3 mm diameter semi occlusion is in the figure (I assume it is the cylindrical part from about 16.5 mm to 18.5 mm on the lower plot) and also what the vertical line is indicating on the lower plot.
Response: The lip tube begins at 35 cm. The 16.8-18.5 cm region is the epilaryngeal airway. More clarity has been provided, lines 331-335

L. 374: ‘e’ missing on ‘the’.
Response: Done

L. 380: Is there a source to back up the statement ‘It is believed …’?
Response: The statement has been modified to be based on the maximum power transfer theorem, lines 437 - 439

L. 399: Can the ‘variable enhancement above 2000 Hz’ comment be supported?
Response: Reference is made to the gaps in the inertagram, line 458

L. 404: As a practical conclusion for singers, could this be dimensioned in terms of what sort of adjustment extents would be appropriate to cover different sound qualities and intensities?
Response: A range of cross-sectional areas and a reference are provided , lines 464 - 465

L. 571: I am not sure what the message is here – are you advocating the avoidance of pressed voice or of pressed voice with reduced VF contact or suggesting the motor sensory memory needs to ‘learn’ to avoid it?

Response: Sentence is revised to clarify contact pressure, lines 632 -634

Reviewer #2: The manuscript provides a strong and thorough outline with detailed results. However, a few minor revisions would aid in the overall flow the manuscript, especially with respect to the discussion section. First, the methods section should be moved before the discussion section so that the reader understands how the results which are being discussed we obtained. Second, a statistical analysis, or argument as to why in this situation it is not needed, would be useful to create a more compelling argument with the data, especially for Table 1. Given the discrepancy in data between the multiple studies cited and the novel data provided, an analysis would be useful to show whether these results agree with each other, and to provide more robustness behind the novel Young's modulus based simulation used. If the author' feel that this is not needed for what is being argued, a more explicit reason should be provided to explain the variation in the expansion of the vocal tract and whether this variation is reasonable given the various experiments. This could be done with a single sentence or two, which is why it is felt that only minor revisions are needed.

Response: We had purposely moved the Methods section behind the Results because that appears to be the more common organization in PLOS publications. Neither of the other reviewers seemed to object, so we decided to leave it as is. We have addressed the problem with doing formal statistics on the measured data, lines 287 - 298. We have also addressed the dependency of the simulation results on the Young’s modulus of the airway walls, lines .

Reviewer #3: General comments

Thank you so much for allowing me to review this very interesting manuscript. I think it is definitely a nice contribution to the field and adds new content to a relevant topic in voice training and therapy: the semioccluded vocal tract exercises. The paper is appreciated for its attention to many details in research design and study execution. Some minor additions I think could contribute to the paper.


Introduction

Could authors provide to readers a brief explanation about why aerodynamic and acoustic interaction between the source and the airway play a major role when the sound source is located deep within the airway? Why is it more efficient?

Response: Three new sentences have been provided, lines 84 - 87

Line 82: is there evidence that musical wind instruments exhibit the same interaction phenomena? I mean, is there evidence for instance that lips vibrate in an easier way because of the long trumpet tube? If so, could authors provide some references?

Response: A reference is provided from trumpet playing, line 89

Line 87: what do you mean by optimal sound production? More efficient in terms of energy conversion? More economic? Please briefly explain this.

Response: Optimal has been replaced with less effortful and more efficient, lines 93 - 94

Line 103-105: Could you please mention (in parentheses) that you are talking about acoustic-aerodynamic interaction and mechano-acoustic interaction respectively?

Response: More clarity between the two types of interaction has been given, lines 115 – 117

Line 106-107: Some references are needed to support that statement.

Response: A Fant (1960) reference and a qualifying statement for impedance at resonance has been added, lines121 - 122

Line 112-113: Statement about ear channel needs some references.

Response: A reference has been given, line 127

Results and discussion

Line 180: Since in clinic lung pressure is usually measure in cm of H2O, could authors provide the amount of H2O equivalent to 2.5 kPa?

Response: The pressure in H2O has been added, line 204

Why only a 3 mm tube was used for airway expansion? Please provide a brief explanation. Response: Four different diameters were tested, as shown in later figures. We show the 3 mm case in Fig. 3 as an example

Line 206-208: “Note that the wider oral tubes (5 mm and 6 mm) produced only a modest glottal expansion, whereas the 3 mm tube dramatically separated the vocal folds at glottal entry and exit” Is this caused by the greater oral pressure generated by thinner tubes? If so, provide a brief explanation to readers.

Response: Yes, greater oral pressure will produce greater airway expansion An entire paragraph is added to explain this, lines 242 - 246

Line 348: Tube used in Guzman et al. 2013 and Guzman et al. 2017 differ in inner diameter. I think, Guzman et al. 2013 used narrow straw and Guzman et al. 2017 used regular drinking straw (wider inner diameter). Could this be an additional explanation for the epilarygeal tube configuration (narrower) reported during tube phonation in Guzman et al. 2013? Maybe the epilarygeal tube narrowing reported by Guzman et al. 2013 was a reaction of the source system, specifically of the epilaryngeal airway resistance (Re) (active narrowing) to match the resistance at the lips (narrow straw) in order to increase the maximum power transfer. Since the resistance at the lips was lower (wider straw) in Guzman et al. 2017, maybe the source system, specially Re did not react in the same way (no active narrowing) because there was no need to get narrower to increase the power transfer. What do you think about this? It would be interesting to include this at this part of the manuscript or in the discussion section.

Furthermore, in a laryngoscopic study by Guzman et al. 2013, it was also found that the degree of airflow resistance at the lips affect the degree of anterior-posterior laryngeal compression (i.e. epilaryngeal tube narrowing). Phonation into a thin straw and tube submerged 10 cm into the water produced the greatest degree of epilaryngeal tube narrowing compared to a wider straw and other SOVTE.
Peltokoski et al, 2015 found similar results in a laryngoscopic study. They reported a narrower epilaryngeal tube during phonation in water (high resistance) compared to tube phonation in air (lower resistance). I think this could be a nice discussion. What do authors think?

Response: We deeply appreciate the interpretations and supplementary data provided by the reviewer concerning the Guzman and Peltokoski papers, but we respectfully wish to avoid further speculation about agreement or disagreement of results.

Line 377-380: “As the epilaryngeal airway resistance is increased with active narrowing, the glottal resistance is reduced by the increase in passive pressure from the supraglottal airway, spreading the vocal folds apart and thereby reducing Rg.”
Regarding this paragraph, I assume that the model implemented in this study did not include a possible compensatory muscle and aerodynamic behavior from the subglottis. It has been previously observed that when there is an increment in oral pressure caused by high airflow resistance voice exercises, there is an automatic compensation, an increment of the subglottic pressure (e.g. Radolf et al. 2014, Guzman et al. 2015), which in turn, tend to increase vocal folds adduction in some cases. If the increment of subglottic pressure is greater than the increment of oral pressure, then the transglottal pressure will increase. On the other hand, if the oral pressure increment is greater than the subglottic pressure, the transglottal pressure will decrease. Therefore, it could be the case that in human subjects the increment of epilaryngeal airway resistance with active narrowing, not necessarily produces a reduced glottal adduction by spreading the vocal folds apart. It could also happen that this compensatory behavior (increased Psub as a reaction of the increased Poral) is only an immediate automatic reaction, but with some sensory or motor memory this could be properly train not to increase transglottal pression, as authors stated in the conclusion. I think this entire point could also be a nice aspect to discuss in the paper.

Response: Thank you for this comment. Transglottal pressure is not a pressure applied to any airway wall. It is the driving pressure for glottal airflow, not vocal fold tissue displacement. Generally, oral pressure rises in proportion to subglottal pressure in a semi-occluded airway. The tissue driving pressure on the vocal fold surfaces is then roughly the average between subglottal and supraglottal pressures, not the difference between the two.


Conclusion

Line 573: “Use of full lung pressure with semi-occlusions seems to present no problem because greater lung pressure does not produce more vocal fold collision”.
As mentioned above this could occur only if transglottal pressure does not substantially increase during semi-occlusions (because of an increased compensatory Psub). Some studies have reported an increased Ptrans (e.g. Guzman et al. 2015).

Response: See explanation above.

Moreover, an increased transglottal pressure and a subsequent increased vocal folds adduction and collision should not be considered always as a detrimental behavior during SOVTE. In voice conditions such as vocal folds paralysis, presbiphonia, or hypophonia caused by Parkinson Disease, an increased vocal folds impact stress is totally desirable. Previous high-speed digital imaging studies have suggested that high airflow resistance SOVTE tend to produce and increase in vocal folds impact stress (Laukkanen et al. 2007, Guzman et al. 2017)

Response: Thank you for the comment. It seems clear that SOVT exercises may not be efficacious for every voice disorder and that decreased glottal resistance may not always be the answer. Clearly, if the vocal folds have insufficient adduction, maximum power transfer may require increased glottal resistance.

**Have all data underlying the figures and results presented in the manuscript been provided?**
Large-scale datasets should be made available via a public repository as described in the *PLOS Computational Biology* [data availability policy](http://journals.plos.org/ploscompbiol/s/data-availability), and numerical data that underlies graphs or summary statistics should be provided in spreadsheet form as supporting information.

Reviewer #1: Yes

Reviewer #2: None

Reviewer #3: Yes

PLOS authors have the option to publish the peer review history of their article ([what does this mean?](https://journals.plos.org/ploscompbiol/s/editorial-and-peer-review-process#loc-peer-review-history)). If published, this will include your full peer review and any attached files.


If you choose “no”, your identity will remain anonymous but your review may still be made public.


**Do you want your identity to be public for this peer review?** For information about this choice, including consent withdrawal, please see our [Privacy Policy](https://www.plos.org/privacy-policy).

Reviewer #1: No

Reviewer #2: No

Reviewer #3: No

Figure Files:

While revising your submission, please upload your figure files to the Preflight Analysis and Conversion Engine (PACE) digital diagnostic tool, [https://pacev2.apexcovantage.com](https://pacev2.apexcovantage.com/). PACE helps ensure that figures meet PLOS requirements. To use PACE, you must first register as a user. Then, login and navigate to the UPLOAD tab, where you will find detailed instructions on how to use the tool. If you encounter any issues or have any questions when using PACE, please email us at [figures@plos.org](mailto:figures@plos.org).

Data Requirements:

Please note that, as a condition of publication, PLOS' data policy requires that you make available all data used to draw the conclusions outlined in your manuscript. Data must be deposited in an appropriate repository, included within the body of the manuscript, or uploaded as supporting information. This includes all numerical values that were used to generate graphs, histograms etc.. For an example in PLOS Biology see here: <http://www.plosbiology.org/article/info%3Adoi%2F10.1371%2Fjournal.pbio.1001908#s5>.

Reproducibility:

To enhance the reproducibility of your results, PLOS recommends that you deposit laboratory protocols in protocols.io, where a protocol can be assigned its own identifier (DOI) such that it can be cited independently in the future. For instructions see [http://journals.plos.org/ploscompbiol/s/submission-guidelines#loc-materials-and-methods](http://journals.plos.org/plospathogens/s/submission-guidelines)

*In compliance with data protection regulations, you may request that we remove your personal registration details at any time.* [*(Remove my information/details)*](https://www.editorialmanager.com/pcompbiol/login.asp?a=r)*. Please contact the publication office if you have any questions.*
